# Supplementary material for: Short-Term Aerobic Exercise Did Not Change Telomere Length While It Reduced Testosterone Levels and Obesity Indexes in PCOS: A Randomized Controlled Clinical Trial Study
Source: Int J Environ Res Public Health. 2021 Oct 27;18(21):11274. doi: 10.3390/ijerph182111274 (PMC8582753; doi:10.3390/ijerph182111274)
Supplement: Supplementary file 1 [file ijerph-18-11274-s001.zip › ijerph-1432642-supplementary.pdf]

International Journal of
Environmental Research and Public Health

MDPI

Table S1. Protocols for continuous and intermittent aerobic physical training.

		CAT	IAT		
**Week**	**Duration**	**Intensity (HRmax, %)**	**Series**	**Recovery Intensity (HRmax, %) (3 min.)**	**Upper Intensity (HRmax, %) (2 min.)**
1	30	65	6	60	70
2	30	65-70	6	60	75
3	30	70	6	60	80
4	35	70	7	60	80
5	35	70-75	7	65	85
6	35	70-75	7	65	85
7	40	70-75	8	65	85
8	40	70-75	8	65	85
9	40	75	8	65-70	85
10	40	75	8	65-70	85
11	45	75	9	65-70	85
12	45	75	9	65-70	85
13	45	75-80	9	65-70	85-90
14	50	75-80	10	65-70	85-90
15	50	75-80	10	65-70	85-90
16	50	75-80	10	65-70	85-90

* CAT, Continuous Aerobic Training, IAT, Intermittent Aerobic Training; HRmax, Maximum heart rate. There were added five minutes of warming up and five minutes of cool down.

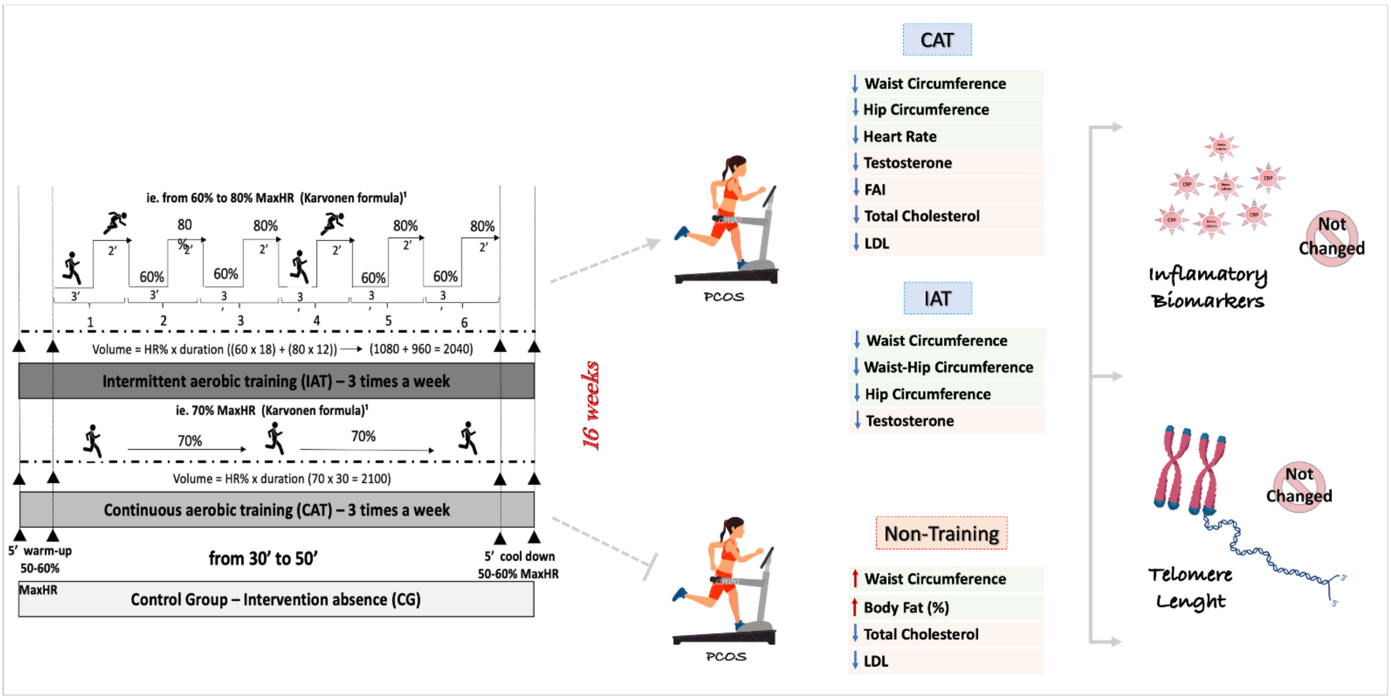

Figure S1. Continuous (CAT) and intermittent aerobic training (IAT) were carried out on a treadmill three times per week (wk) for 16 weeks, lasting equally and progressively from 30 min in 162 the first week, to 50 min in the last week**.** For both protocols, five min warmups and five min cool downs, between 171 50% and 60% of the maximum heart rate, were included. The non-training PCOS control group was evaluated before and after 16 weeks of protocol training. Booth exercises reduced testosterone level. CAT reduced waist (WC) and hip circumference (HC), heart rate, free androgen index, total and LDL cholesterol and IAT reduced WC, HC and waist-hip circumference. The non-training group showed an increase in WC and body fat percentage, and a reduction in total and LDL cholesterol. Inflammatory biomarkers and telomere length did not change after 16 wks of aerobic training or the observational period.
